# Identification and validation of potential biomarkers for atrial fibrillation based on integrated bioinformatics analysis

**DOI:** 10.3389/fcell.2023.1190273

**Published:** 2024-01-11

**Authors:** Fei Tong, Zhijun Sun

**Affiliations:** Department of Cardiology, Shengjing Hospital of China Medical University, Shenyang, China

**Keywords:** atrial fibrillation, bioinformatics analyses, MPV17, HIF1AN, weighted gene co-expression network analysis

## Abstract

**Background:** Globally, the most common form of arrhythmias is atrial fibrillation (AF), which causes severe morbidity, mortality, and socioeconomic burden. The application of machine learning algorithms in combination with weighted gene co-expression network analysis (WGCNA) can be used to screen genes, therefore, we aimed to screen for potential biomarkers associated with AF development using this integrated bioinformatics approach.

**Methods:** On the basis of the AF endocardium gene expression profiles GSE79768 and GSE115574 from the Gene Expression Omnibus database, differentially expressed genes (DEGs) between AF and sinus rhythm samples were identified. DEGs enrichment analysis and transcription factor screening were then performed. Hub genes for AF were screened using WGCNA and machine learning algorithms, and the diagnostic accuracy was assessed by the receiver operating characteristic (ROC) curves. GSE41177 was used as the validation set for verification. Subsequently, we identified the specific signaling pathways in which the key biomarkers were involved, using gene set enrichment analysis and reverse prediction of mRNA–miRNA interaction pairs. Finally, we explored the associations between the hub genes and immune microenvironment and immune regulation.

**Results:** Fifty-seven DEGs were identified, and the two hub genes, hypoxia inducible factor 1 subunit alpha inhibitor (*HIF1AN*) and mitochondrial inner membrane protein MPV17 (*MPV17*), were screened using WGCNA combined with machine learning algorithms. The areas under the receiver operating characteristic curves for *MPV17* and *HIF1AN* validated that two genes predicted AF development, and the differential expression of the hub genes was verified in the external validation dataset. Enrichment analysis showed that *MPV17* and *HIF1AN* affect mitochondrial dysfunction, oxidative stress, gap junctions, and other signaling pathway functions. Immune cell infiltration and immunomodulatory correlation analyses showed that *MPV17* and *HIF1AN* are strongly correlated with the content of immune cells and significantly correlated with *HLA* expression.

**Conclusion:** The identification of hub genes associated with AF using WGCNA combined with machine learning algorithms and their correlation with immune cells and immune gene expression can elucidate the molecular mechanisms underlying AF occurrence. This may further identify more accurate and effective biomarkers and therapeutic targets for the diagnosis and treatment of AF.

## 1 Introduction

Atrial fibrillation (AF) is the most prevalent age-associated arrhythmias occurring in approximately 8.5% of patients aged >65 years, and the lifetime AF risk is estimated to be 1 in 3 individuals of European ancestry (Virani et al., 2021). AF substantially affects patients’ quality of life and confers the risk of morbidity and mortality through stroke and heart failure. In addition to conventional risk factors such as age, hypertension, diabetes, male gender, obesity, obstructive sleep apnea, and endurance exercise (Myrstad et al., 2014; Schnabel et al., 2015; Virani et al., 2021), a hereditary component for AF risk has also been well recognized (Lubitz et al., 2010). The mechanisms underlying AF are not comprehensively clear, and thus, currently available therapeutic options, such as antiarrhythmic medications and catheter ablation procedures, have limited efficacy and adverse effects (Prystowsky et al., 2015). Emerging gene therapy for AF rhythm control may provide a novel and promising approach to AF (Farraha et al., 2016; Yoo et al., 2020). A preliminary genome-wide association study (GWAS) meta-analysis identified at least 134 genetic loci significantly associated with AF risk (Roselli et al., 2018) and increased our understanding of the pathophysiology of AF; however, much of the heritability of AF has been uncharted or inconclusive thus far (Christophersen and Ellinor, 2016). Therefore, there is a growing interest in developing new therapeutic approaches and exploring effective prognostic models for screening patients with high-risk AF.

With the continuous improvement of high-throughput technologies, several bioinformatics databases have been developed, which provide new perspectives for researchers to explore biomarkers and underlying mechanisms of diseases more precisely and effectively. However, traditional experimental analyses require long-term exploration due to the large amount of data, and the accuracy of bioinformatics analysis is influenced by duplicate data and covariance in database. Weighted gene co-expression network analysis (WGCNA) is a bioinformatics method that aggregates genes with the same or similar expression patterns into a module and analyzes each module in association with phenotypic data to identify potential key genes (Langfelder and Horvath, 2008). WGCNA can identify genes that may have great influences on disease development and is thus widely used to identify disease biomarkers. Machine learning has also garnered increasing interest in the screening of disease biomarkers (Wang et al., 2022). Support vector machine based recursive feature elimination (SVM-RFE) is a machine learning algorithm that ranks different genes or features based on the sum of squares of feature coefficients, minimizing empirical errors and thus effectively screening genes with significant impact (Duan et al., 2005). Least absolute shrinkage and selection operator (LASSO) is another machine learning algorithm that fits a generalized linear model while performing variable selection and regularization, which can effectively reduce the effect of covariance and thus screen genes with a significant association between variables (Duan et al., 2016). The application of the two machine learning algorithms SVM-RFE and LASSO, in combination with WGCNA, can effectively avoid identical or similar data as well as covariance and thus screen the hub genes from transcriptome data. However, their application in screening for potential AF biomarkers is rarely reported.

In our study, we aimed to screen for key biomarkers potentially related to the development of AF. We also investigated the potential molecular mechanisms of these biomarkers and their association with immune cell infiltration. We screened differentially expressed genes (DEGs) and related pathways between AF and sinus rhythm (SR) samples using two AF microarray datasets retrieved from the Gene Expression Omnibus (GEO) database. WGCNA and the two above-mentioned machine learning algorithms were applied to screen key biomarkers, and the results were validated using an external validation dataset. Subsequently, single-sample gene set enrichment analysis (ssGSEA) was used to identify the specific signaling pathways in which the key biomarkers were involved. We also quantified the percentages of 28 immune cells in the AF samples and SR samples respectively and the subsequent association between identified biomarkers and infiltrating immune cells. These processes may inform studies on the pathogenesis of AF and the development of new immunotherapeutic targets.

## 2 Materials and methods

### 2.1 Data collection

**FIGURE 1:**
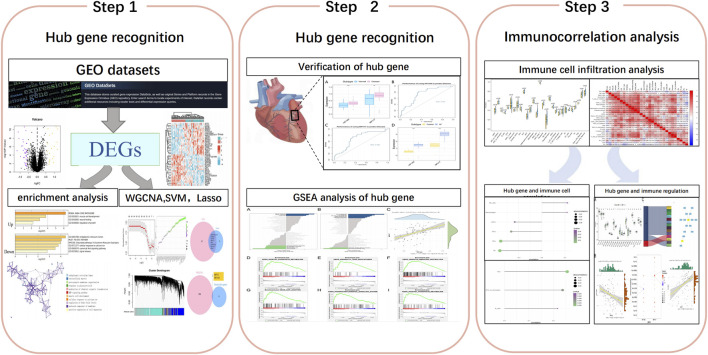
Flowchart.

The workflow of this study is shown in Figure 1. In this study, the microarray datasets were downloaded from the GEO database (https://www.ncbi.nlm.nih.gov/geo/), including GSE79768, GSE115574 and GSE41177. GSE79768 and GSE115574 databases as training set contained the gene data of 42 atrial samples from patients with AF and 43 atrial samples from patients with SR, while GSE41177 database as the validation set included 32 left atrial samples of AF and 6 left atrial samples of SR. The “sva” package in R was used to correct the data between chips for subsequent analysis. All data analyzed in our study were achieved from public databases and thus did not require ethics committee approval.

### 2.2 Enrichment analysis of DEGs and screening of transcription factors

To determine the biological functions and signaling pathways in disease progression, DEGs were annotated and visualized by Metascape (www.metascape.org), and Gene Ontology (GO) analysis was applied for specific genes. A minimum overlap of ≥3 and *p* ≤ 0.01 were regarded as statistical significance. We explored transcription factors based on the DEGs to elucidate the potential molecular mechanisms by which these genes affect AF development. We extracted upstream motifs based on the DEGs, determined the normalized enrichment score (NES), and performed enrichment analysis for each motif by cumulative recovery curves. The R package RcisTarget was applied to predict relevant transcription factors. We used the rcistarget. hg19. motifdb.cisbpont.500bp base to access the gene-motif ranking database.

### 2.3 Construction of machine learning-based models

The LASSO regression algorithm and the SVM-RFE algorithm were used to identify the diagnostic biomarkers for diseases. First, we implemented LASSO regression using the “glmnet” R package to filter and visualize the DEGs. Then, we constructed SVM-RFE models using the “e1071”package to further identify the biomarkers for diseases. Finally, we integrated the screened characteristic genes to obtain optimal characteristic genes, using a Venn diagram.

### 2.4 WGCNA

WGCNA is a systematic biology approach to characterize gene association between patients with SR and those with AF, and to identify highly co-varying gene sets and potential biomarker genes or therapeutic targets based on the endogeneity of gene sets. Transcriptome datas were read and imported using the R package “WGCNA” (http:/www.r-project.org/), followed by which the “hclust” function was used to analyze the dataset for any significant outliers. A soft threshold filter was used to make the constructed network compatible with the characteristics of a scale-free network. Next, the weighted adjacency matrix was transformed into a topological overlap matrix (TOM) to assess the connectivity in the network, and a clustering tree structure of TOM was constructed using a hierarchical clustering method. Different branches of the clustering tree represent different gene modules by different colors according to the weighted correlation coefficients of genes. The conservativeness of the modules was evaluated based on the Z-summary score. In our study, the input WGCNA module genes were crossed with optimal characteristic genes from the machine learning to obtain the hub genes.

### 2.5 Identification and validation of hub genes

Box-line plots were used to assess the expression levels of the identified the hub genes in AF and SR (control) groups respectively. Subsequently, receiver operating characteristic (ROC) curves were used to assess the diagnostic efficacy of the hub genes by “pROC” package. An external database of GSE41177 was employed to validate the hub genes identified in the training set by the Wilcoxon test.

### 2.6 Gene set enrichment analysis (GSEA)

GSEA was used to assess the distribution of the hub genes in a gene table ranked by disease relevance to determine their contribution to disease development by examining whether the hub genes are enriched at the top or bottom of the table. In this study, GSEA was used to compare the differences in signaling pathways between the high- and low-expression groups and to explore the potential molecular mechanisms underlying the discrepancies of AF and SR groups, where the number of substitutions was set to 1000 and the type of substitution was set to phenotype.

### 2.7 Immune cell infiltration and immune correlation analyses

To further understand the status of infiltrating immune cells in these two groups, we analyzed the RNA-sequencing data of these two groups, using ssGSEA, which inferred the relative proportion of infiltrating immune cells. The interaction between 22 immune cell types was then analyzed by the “corrplot” package to further display the interaction of immune cells. Furthermore, the relative extent of immune cell infiltration was plotted by the “vioplot” package to evaluate the effect of the hub genes on immune infiltration. Spearman correlation analysis was performed for gene expression as well as the extent of immune cell infiltration.

### 2.8 Statistical analysis

Statistical analyses were performed using R (version 3.6). All tests were two-sided with a significance level of *P* of <0.05.

## 3 Results

### 3.1 Identification of DEGs

**FIGURE 2:**
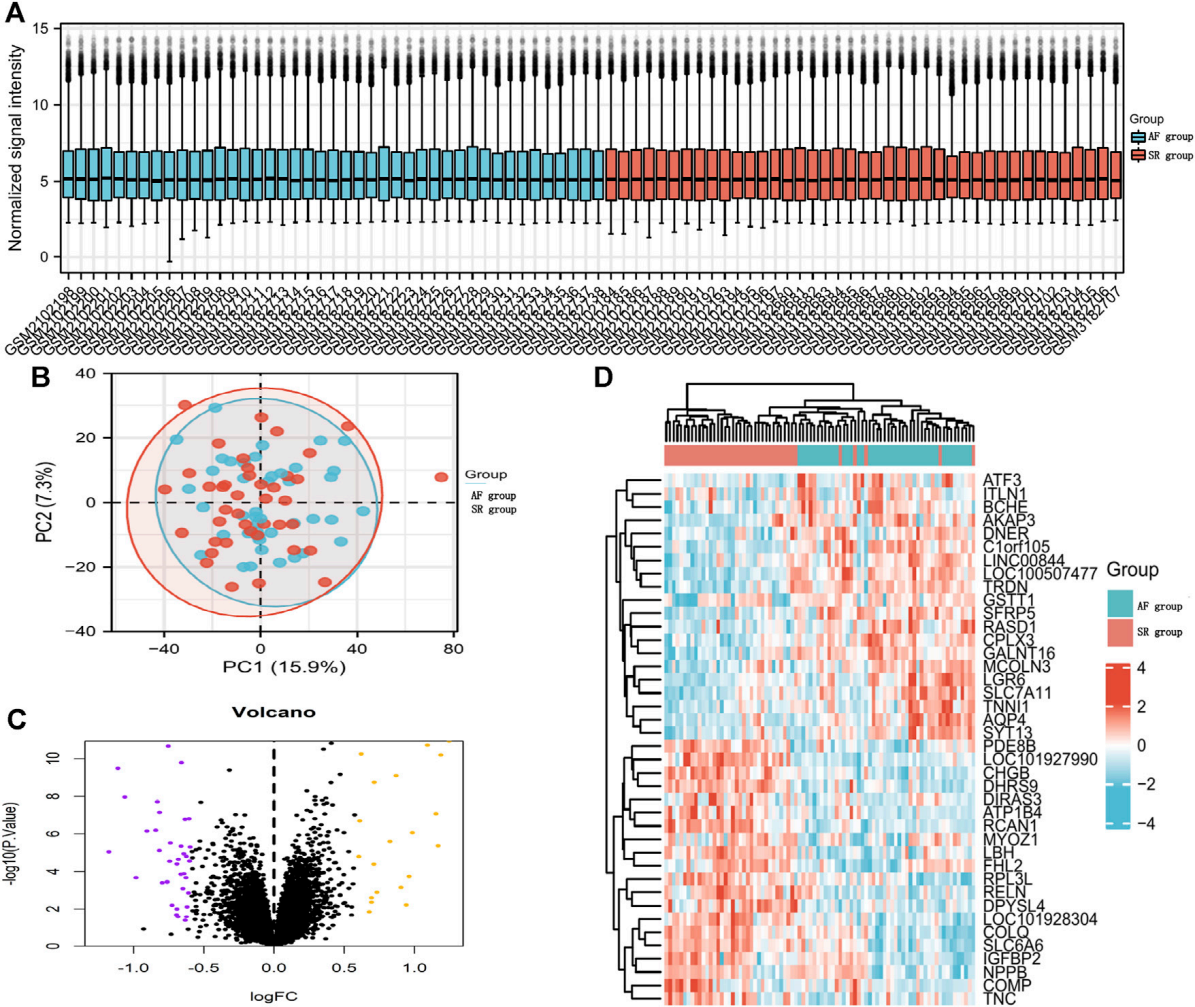
DEGs between AF patients and SR patients. **(A)** Boxplot diagram of the DEGs between the two groups. **(B)** Principal component analysis between the two groups. **(C)** Volcano plot visualizing DEGs between the two groups. **(D)** Heatmap displaying DEGs between the two groups.

We downloaded GSE79768 and GSE115574 AF datasets from GEO and divided 85 patients into SR (n = 43) and AF (n = 42) groups. After merging microarray data, the batch effects were adjusted by the “sva” package in R. Normalization was displayed by a boxplot (Figure 2A) and clustering between sample subgroups was displayed by a principal component analysis plot (Figure 2B). DEG analysis was performed by the linear model (limma) package in R, and 57 DEGs (20 upregulated and 37 downregulated) with *P* of <0.05 and fold change (FC) of ≥0.58 were displayed by a volcano plot in Figure 2C. In addition, the top 20 upregulated DEGs *versus* the top 20 downregulated DEGs were shown in the heat map in Figure 2D.

### 3.2 Functional enrichment analysis of DEGs and screening of transcription factors

**FIGURE 3:**
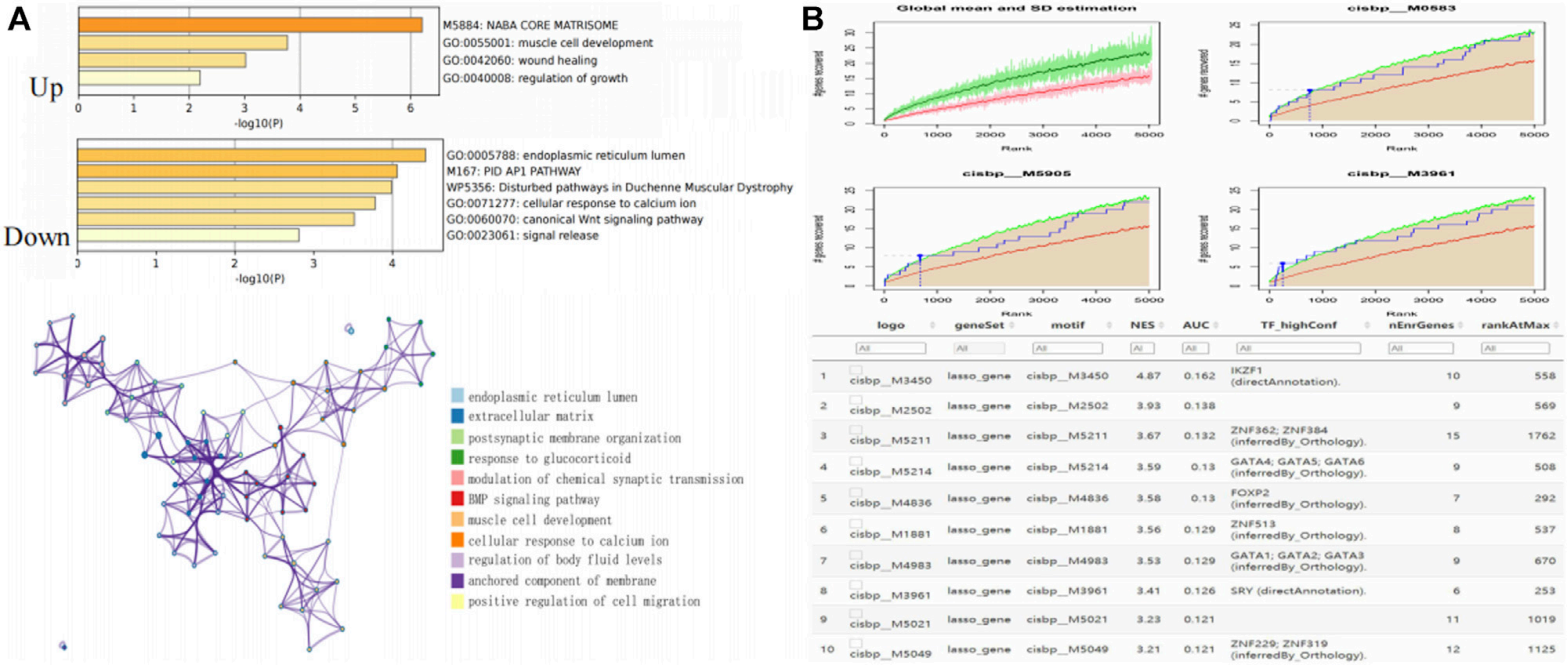
Enrichment analysis and transcriptional regulation analysis of DEGs. **(A)** Enrichment analysis among DEGs. **(B)** Transcriptional regulation analysis among DEGs.

Metascape analysis indicated that the upregulated DEGs and downregulated DEGs were mostly enriched in the core matrisome pathway and endoplasmic reticulum lumen pathway respectively. These results suggested that different mRNAs fulfilled different physiological functions and were involved in the regulation of AF (Figure 3A). We further explored the downstream regulatory mechanisms of the DEGs and found them to be regulated by multiple transcription factors via a common mechanism. Therefore, we implemented an enrichment analysis of these transcription factors through cumulative recovery curves and Motif-TF annotation and then screened for important genes. The transcription factor of IKZF1 annotated as a motif of cisbp_M3450 was the main regulator in the gene set; 10 DEGs were enriched in this motif, and the NES was 4.87. The results showed that all the motifs and corresponding transcription factors were enriched in 10 DEGs (Figure 3B).

### 3.3 Construction of machine learning models

**FIGURE 4:**
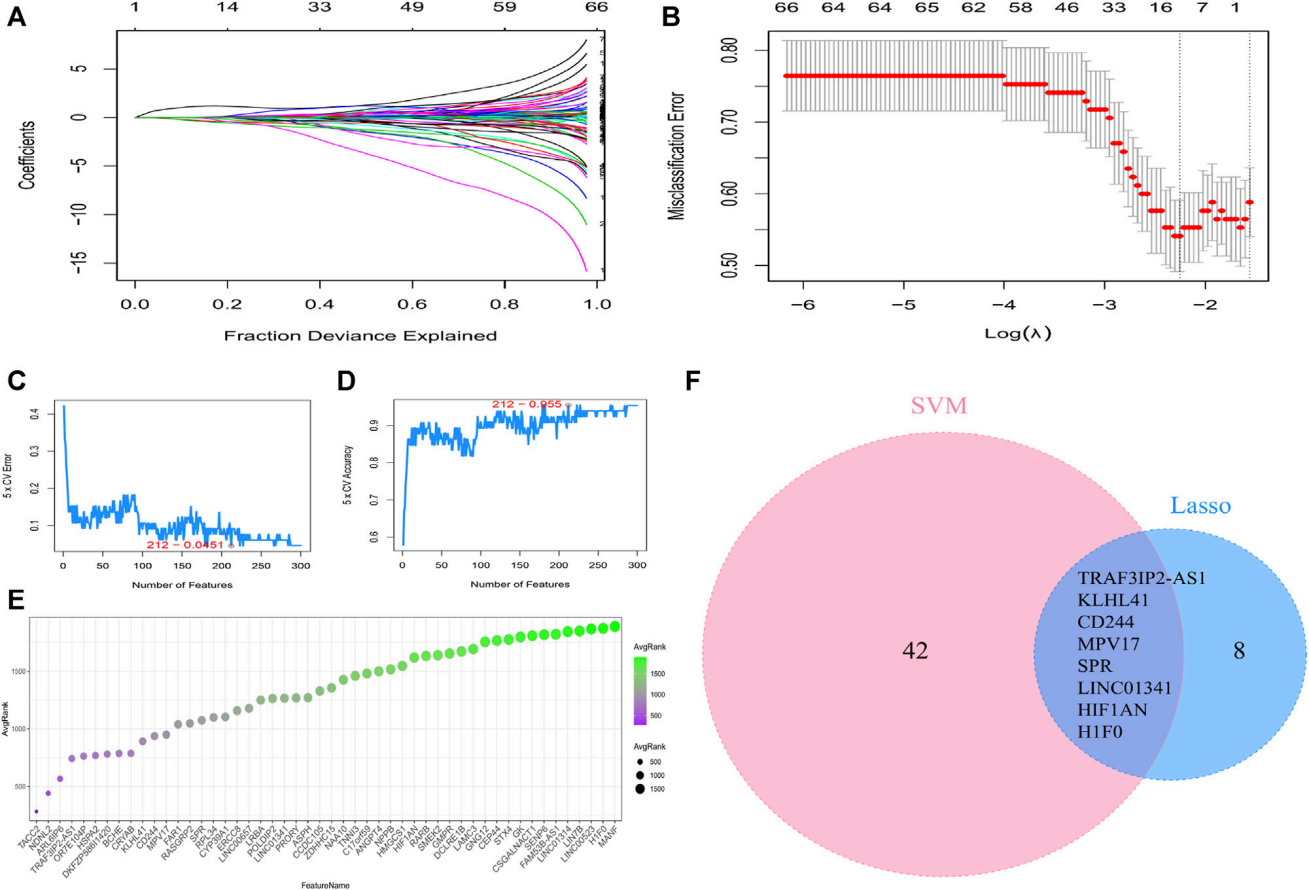
Machine learning screening of the characteristic genes. **(A)** LASSO coefficient curves. **(B)** Determination of the optimal penalty coefficient (lambda) and the minimum absolute shrinkage criterion in the Lasso regression. **(C)** Error rate of genes identified by SVM-RFE machine learning. **(D)** Accuracy rate of genes identified by SVM-RFE machine learning. **(E)** The top 50 genes with the highest accuracy by SVM-RFE. **(F)** Venn diagrams to identify the characteristic genes based on LASSO and SVM-RFE screening.

LASSO regression and SVM-RFE were performed to identify the characteristic genes that were most relevant to AF. LASSO identified 16 genes of AF (Figures 4A, B). 50 genes with the highest accuracy screened by SVM-RFE (Figures 4C–E) took the intersection with the genes identified by LASSO regression, and 8 characteristic genes of AF were ultimately identified (Figure 4F).

### 3.4 Construction of Co-Expression modules and identification of the hub genes

**FIGURE 5:**
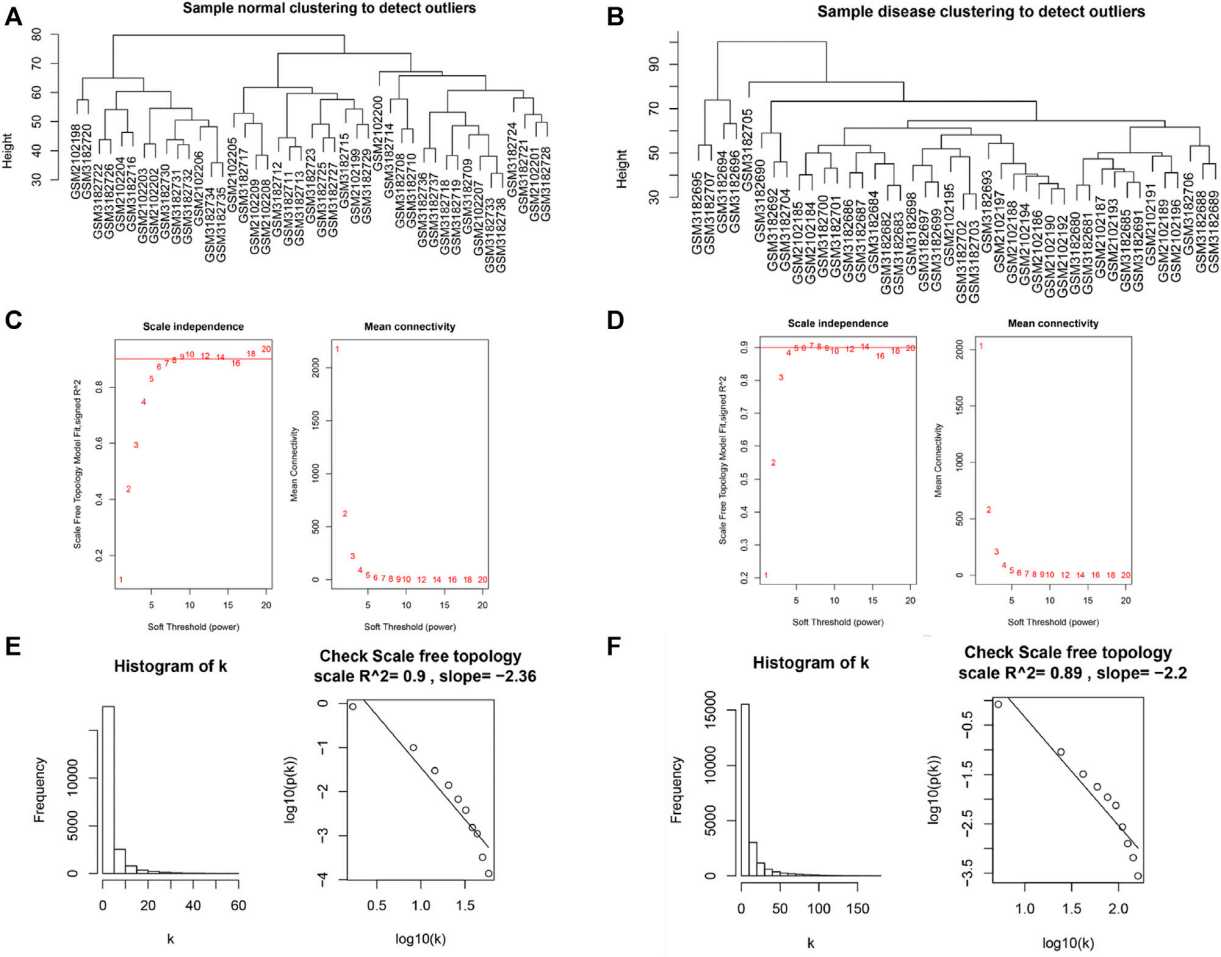
Verification of WGCNA feasibility. **(A)** The clustering dendrogram of SR patient samples to detect outliers. **(B)** The clustering dendrogram of AF patient samples to detect outliers. **(C)** Scaleless fit index and average connectivity of soft threshold power of SR patients. **(D)** Scaleless fit index and average connectivity of soft threshold power of AF patients. **(E)** Histogram of k and correlation coefficient between k and p (k) of SR patient samples. **(F)** Histogram of k and correlation coefficient between k and p (k) of AF patient samples.

AF is a heterogeneous syndrome, and multiple modulating genes are involved in its pathogenesis. In most diseases, genes with similar expression patterns are prone to have similar biological functions. Thus, gene co-expression networks can facilitate analysis of AF-related biological pathways. We included all genes from 85 patients and found no significant outlier samples in the dataset using the hclust function (Figures 5A, B). A soft threshold power was selected to make the constructed network compatible with the characteristics of scale-free networks. We set the soft threshold to 9 in SR group (Figure 5C) and 7 in AF group (Figure 5D). Through the calculation of the scale-free topology, the *R* ^2^ value reached 0.9 (Figures 5E, F). These results further verified the feasibility of WGCNA.

**FIGURE 6:**
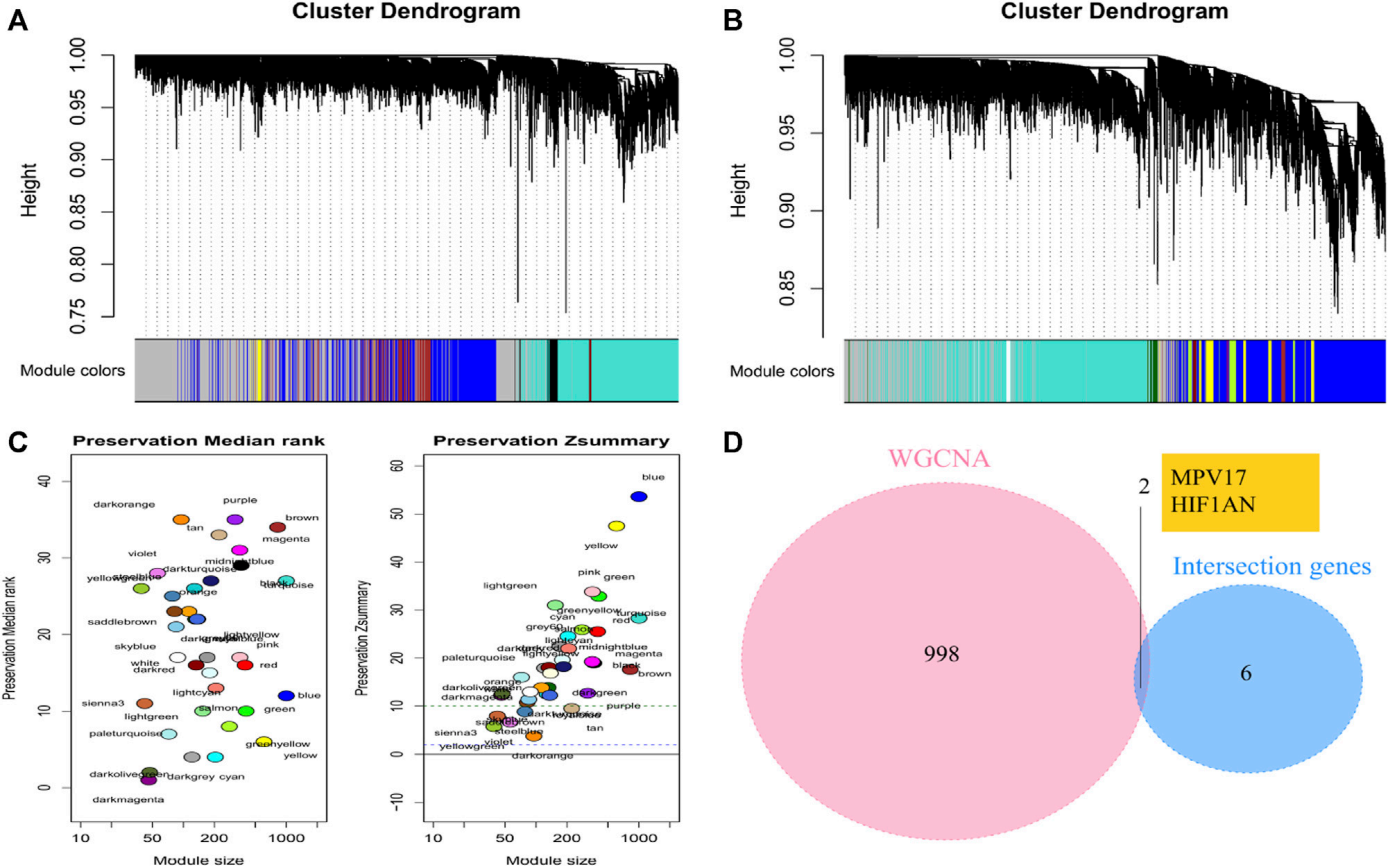
Identification of trait-module genes by WGCNA **(A)** Clustering dendrograms of SR samples. **(B)** Clustering dendrograms of AF samples. **(C)** The preservation median rank and Z summary score of co-expression modules. **(D)** Venn diagrams to identify the hub genes based on WGCNA and machine learning models.

Two co-expression networks of genes from 85 patients were constructed and hierarchical clustering analysis was performed according to weighted correlation coefficients. The clustering results were segmented based on set standards to obtain different gene modules (Figures 6A, B). We identified 35 modules of different sizes represented by the branches of the cluster tree with different colors by WGCNA for the AF group. The network modules in SR group were compared with those in AF group to identify non-conserved modules that could be interpreted as the change in the network attributed to AF group. These non-conserved modules might be related to disease progression in patients with AF. The median rank and Z-summary scores of conservatism of different color modules were shown in Figure 6C. The Z-summary score of the blue module was the highest, indicating that it retained the network characteristics of SR group. The dark orange module with the lowest Z-summary score was less conservative, indicating that it could be used as a module feature to distinguish patients with AF from those with SR. We extracted the first 1000 genes of the module and took the intersection with 8 characteristic genes screened through LASSO and SVM, and finally obtained two hub genes: hypoxia inducible factor 1 subunit alpha inhibitor (*HIF1AN*) and mitochondrial inner membrane protein MPV17 (*MPV17*) (Figure 6D). And the enrichment analysis for other genes included in the dark orange module was displayed in Supplementary Figure S1.

### 3.5 Identification and validation of hub gene expression levels and diagnostic value

**FIGURE 7:**
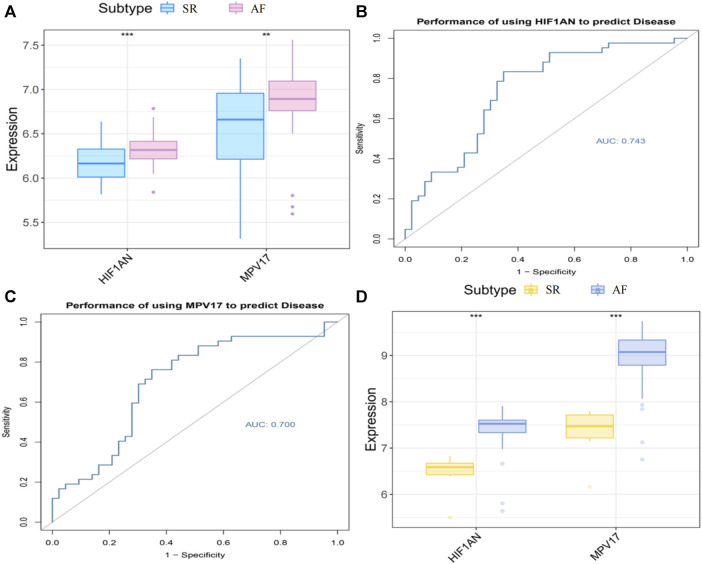
The hub gene expression in AF and SR. **(A)** Expression levels of the hub genes in two groups. **(B)** ROC curve of *HIF1AN* in AF group. **(C)** ROC curve of *MPV17* in AF group. **(D)** Hub genes expression in the external validation database GSE41177. Statistical test: * *p < 0.01, * * *p < 0.001.

The expression levels of *HIF1AN* and *MPV17* were assessed by box-line plots and found to be significantly higher in AF group than those in SR group (*p* < 0.001) (Figure 7A). The sensitivity and specificity of two hub genes as diagnostic genes were evaluated by the area under the ROC curve (AUC). The AUCs of *HIF1AN* and *MPV17* were 0.743 (Figure 7B) and 0.700 (Figure 7C), respectively, which indicated that *HIF1AN* and *MPV17* had high diagnostic value and could better predict disease development. To further validate the results of the bioinformatics analysis, we downloaded GSE41177 dataset from GEO database, which included 32 cases with AF and 6 cases with SR and verified the significant differences in the expression of *HIF1AN* and *MPV17* between AF and SR by Wilcoxon test (*p* < 0.05) (Figure 7D). The enrichment analysis for two hub genes in metabolic processes was displayed in Supplementary Figure S2.

### 3.6 GSEA

**FIGURE 8:**
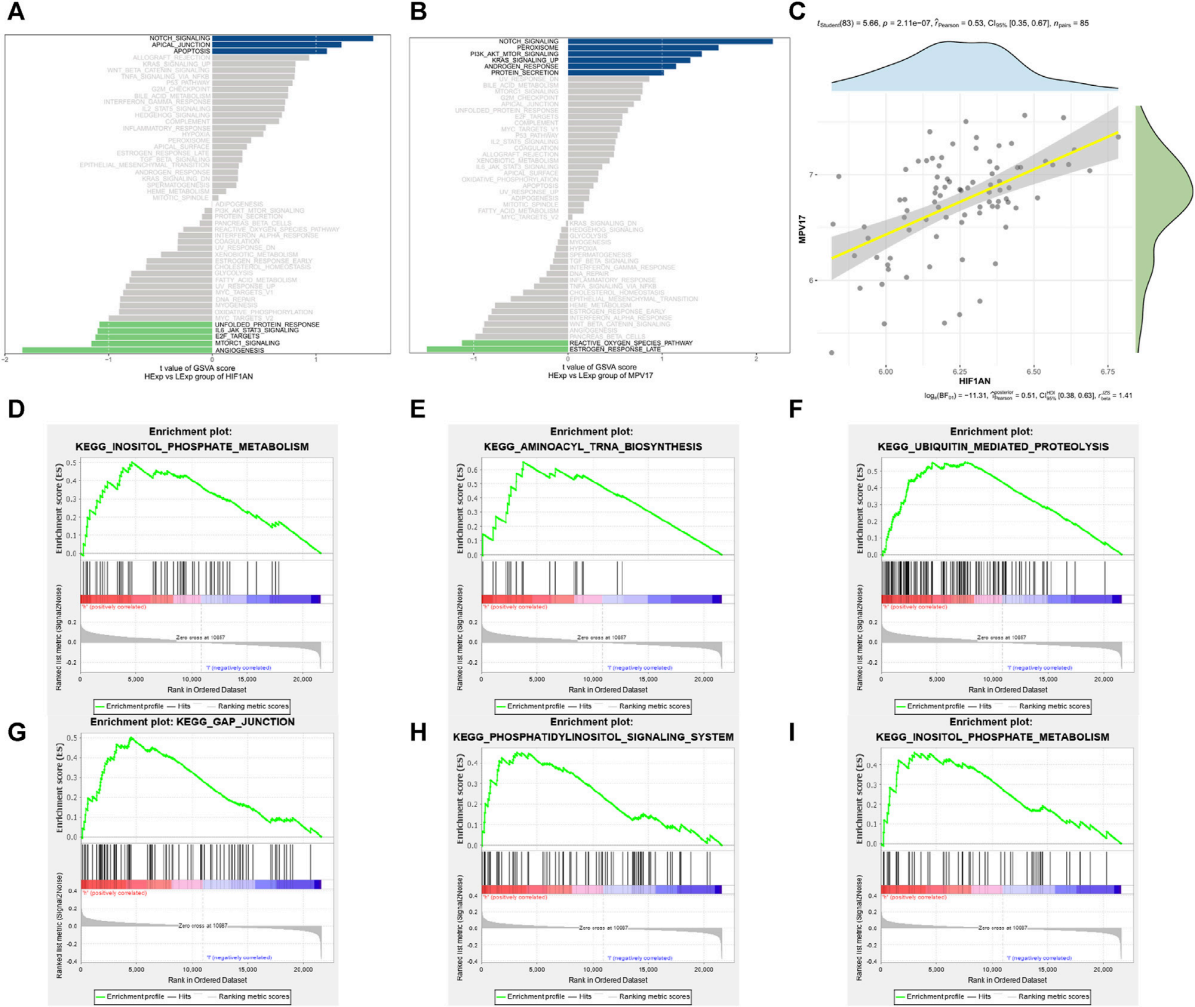
Enrichment pathway of the hub genes. **(A)** The main enrichment pathway of high expression of *HIF1AN*. **(B)** The main enrichment pathway of high expression of *MPV17*. **(C)** Correlation between *HIF1AN* and *MPV17* expression. **(D,E)** The first three enriched pathways of *HIF1AN*: inositol phosphate metabolism **(D)**; aminoacyl trna biosynthesis **(E)**; ubiquitin mediated proteolysis **(F)**. **(G–I)**The first three enriched pathways of *MPV17*: gap junction **(G)**; phosphatidylinositol signaling system **(H)**; inositol phosphate metabolism **(I)**.

The specific signaling pathways in which the two hub genes got involved were explored to reveal the specific molecular mechanisms associated with AF development. As shown in Figure 8A, the main pathways enriched by the high expression of *HIF1AN* were INOSITOL PHOSPHATE METABOLISM (Figure 8D), AMINOACYL TRNA BIOSYNTHESIS (Figure 8E), and UBIQUITIN MEDIATED PROTEOLYSIS (Figure 8F); as shown in Figure 8B, the pathways enriched by high expression of *MPV17* were mainly GAP JUNCTION (Figure 8G), PHOSPHATIDYLINOSITOL SIGNALING SYSTEM (Figure 8H), and INOSITOL PHOSPHATE METABOLISM (Figure 8I). And correlation analysis revealed a positive relationship between *HIF1AN* and *MPV17* expression (Figure 8C).

### 3.7 Immune cell infiltration and correlation analysis between hub genes and immune cells

**FIGURE 9:**
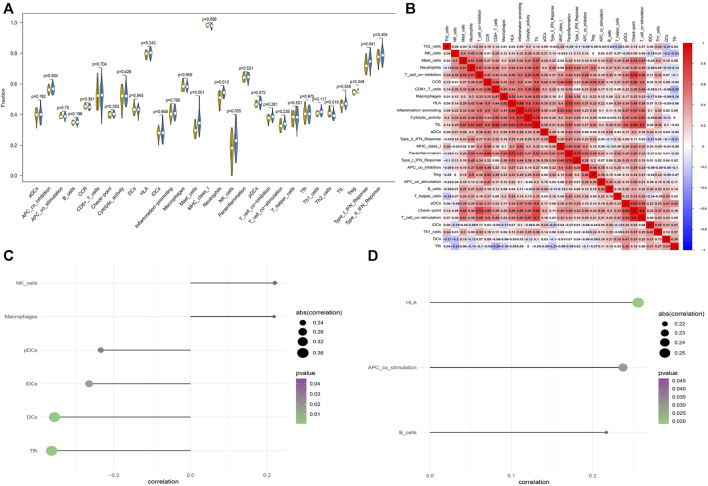
The immune cell infiltration in patients with AF and patients with SR. **(A)** The difference in immune cells between patients with AF and patients with SR. Blue represents patients with AF and yellow represents patients with SR **(B)** Correlation heatmap of immune cells. Red represents positive correlation and blue represents negative correlation. **(C)** The correlation between *HIF1AN* and immune cells. **(D)** The correlation between *MPV17* and immune cells. Statistical test: * *p < 0.01.

By virtue of analysis of the association between hub genes and immune cells in AF dataset, the potential molecular mechanisms by which *HIF1AN* and *MPV17* influence AF development were revealed. CIBERSORT-based immune cell infiltration analysis indicated that mast cells, neutrophils, natural killer (NK) cells, and type I interferon (IFN) were significantly higher in patients with AF and T helper 2 (Th2) cells were significantly lower in AF (Figure 9A). The immune cell correlation was manifested in Figure 9B, in which red represented positive correlation and blue represented negative correlation. The correlation of *HIF1AN* and *MPV17* with immune cells was analyzed and found that both *HIF1AN* and *MPV17* were strongly correlated with quantities of immune cells (Figures 9C,D).

### 3.8 Correlation analysis between hub genes and immune genes

**FIGURE 10:**
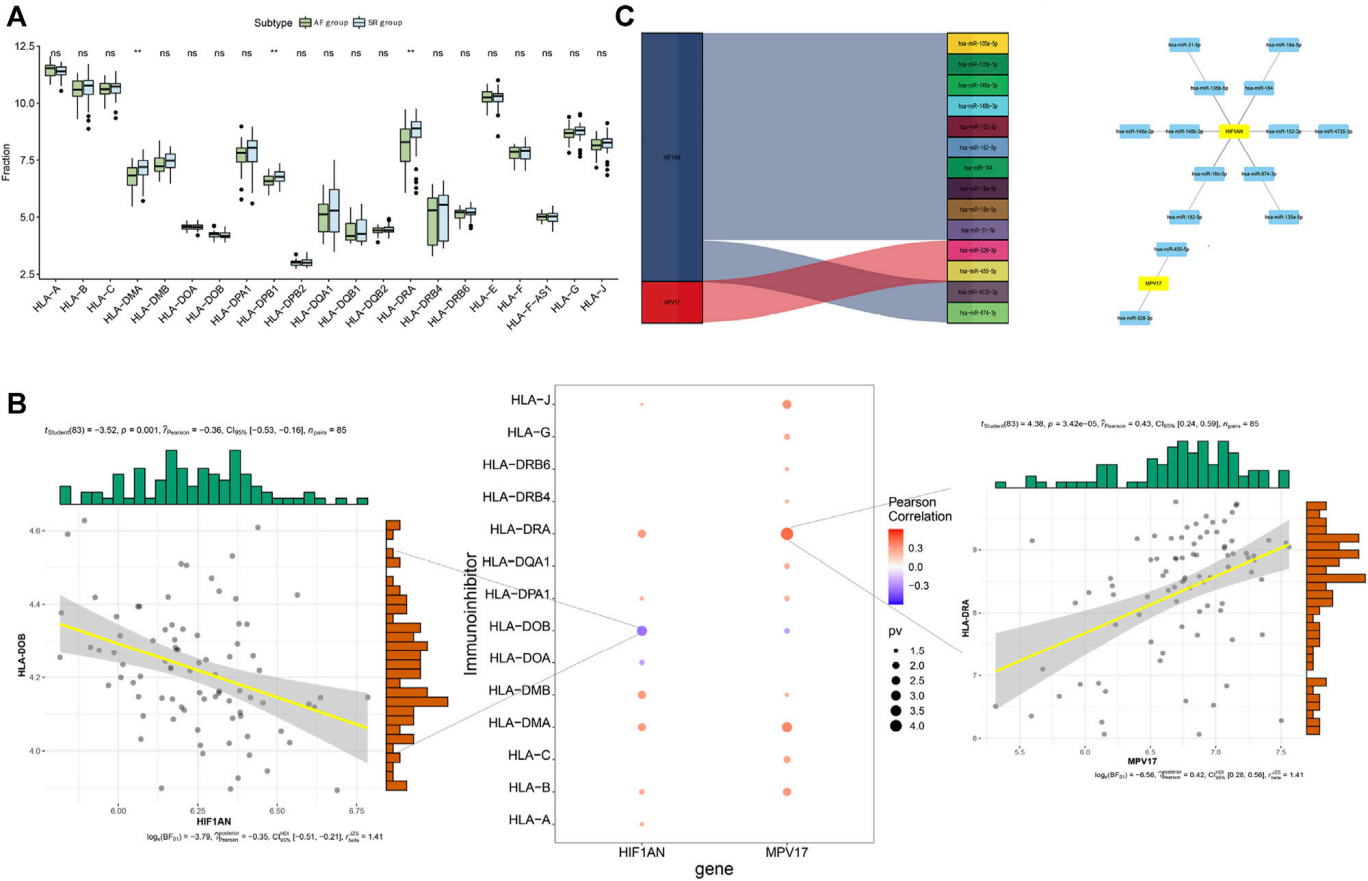
Analysis of immune regulatory genes related to the hub genes and prediction of mRNA-miRNA regulating the hub genes. **(A)** Differential analysis of immune regulatory genes. **(B)** Correlation analysis of the hub genes and immune regulatory genes **(C)** Prediction of mRNA-miRNA regulating the hub genes.

The differential expression analysis of immune regulatory genes indicated that the significantly different levels of *HLA-DMA*, *HLA-DPB1*, and *HLA-DRA* were observed between the AF group and SR group (Figure 10A). The correlation analysis between the hub genes and the related immune regulation genes indicated that *HIF1AN* was significantly negatively correlated with *HLA-DOB* and *MPV17* was significantly positively correlated with *HLA-DRA* (Figure 10B). In addition, reverse-prediction of the two hub genes by FunRich 3.1.3 visualized a total of 14 pairs of mRNA–miRNA interaction (Figure 10C).

## 4 Discussion

AF is a complex disease characterized by environmental and genetic factors that contribute to its pathogenesis. The onset and maintenance of AF require electrical and structural remodeling of the atria (Nattel and Harada, 2014). Atrial electrical remodeling is characterized by shorter effective refractory periods and slowed conduction velocity due to alterations in cardiac action potential, indicating a re-entry substrate in AF (Yeh et al., 2008). Structural remodeling includes atrial dilation, cell hypertrophy, and fibrosis, all of which also contribute to the abnormal electrical signal formation (Allessie et al., 2002). The molecular basis of the mechanisms underlying AF is not completely understood and attributed to fibrosis, calcium dysregulation, inflammation, and oxidative injury (Ferrari et al., 2016; Nattel, 2017). To unravel the mechanism, the genetic variation has increasingly been identified and categorized into three components: rare variation—encoding ion channels and gap junction proteins and influencing cardiac depolarization and repolarization in familial forms of AF; common variation—identified using GWAS and regulating cardiac development and cardiomyocyte contractility and structure; and unidentified variation (Tucker et al., 2016). Genetic variation in AF is heterogeneous. To better understand the pathogenesis and key biomarkers of AF, we compared DEGs between the AF and SR groups based on two GEO datasets and performed enrichment analyses to explore the potential biological functions of DEGs in AF. To more accurately identify central genes associated with AF pathogenesis, we screened two hub genes by combining machine learning algorithms (SVM-RFE and LASSO) based on WGCNA and also confirmed the validity of *HIF1AN* and *MPV17* in both the training and validation sets. Subsequently, using gene enrichment analysis and GSEA, we found that *HIF1AN* and *MPV17* can affect key signaling pathway functions, such as mitochondrial dysfunction, oxidative stress, and gap junctions. Finally, we performed a correlation analysis between the hub genes and immune cell regulation. We attempted to develop a deep understanding of the pathogenesis and key biomarkers of AF through our study and identify the potential targets for early diagnosis and treatment of AF.

We found that the mRNA expression levels of *HIF1AN* and *MPV17* were significantly upregulated in AF samples compared to those with sinus rhythm, and there was a positive correlation between these two genes. Therefore, *HIF1AN* and *MPV17* could be considered diagnostic biomarker for AF.

*MPV17,* located at 2p23-p21 encodes a mitochondrial inner membrane protein associated with mitochondrial homeostasis and reactive oxygen species (ROS) metabolism (Spinazzola et al., 2006; Casalena et al., 2014). In a swine rapid atrial pacing model of AF, increased activity of ROS in both of the left atria and left atrial appendage (Cai et al., 2002). The ROS can cause atrial fibrosis and increase the susceptibility to AF (Friedrichs et al., 2012). As the major source of intracellular ROS, mitochondrial oxidative stress can lead to arrhythmias such as AF through alterations of ion homeostasis and ion channel behavior (Liu et al., 2022). The precise function of *MPV17* in mitochondria has not been established. An enhanced ROS production was registered in *MPV17* gene-inactivated mice (Binder et al., 1999; Casalena et al., 2014). The *MPV17* gene product was identified as a membrane protein of peroxisomes playing a major role in the peroxisomal metabolism of ROS (Zwaka et al., 1994). *MPV17* forms a non-selective channel in the inner mitochondrial membrane to decrease mitochondrial membrane potential that may be beneficial under some conditions to preserve mitochondrial homeostasis by preventing excessive production of ROS (Antonenkov et al., 2015). However, elevated *MPV17* in AF in our bioinformatics-based findings appeared to be counterintuitive, and one possible explaination is that it represents a compensatory mechanism in response to excessive ROS in AF. It has been proposed that mitochondria targeted antioxidants may mitigate the progression of arrhythmia (Liu et al., 2022).

*HIF1AN*, also known as *FIH-1,* is located at 10q24 and encodes a protein that binds to hypoxia inducible factor 1-alpha (*HIF-1α)* to negatively modulate *HIF-1α* stability and inhibit *HIF-1α* signaling (Mahon et al., 2001). The *MPV17* lacking cells displayed enhanced *HIF-1α* signaling under normoxia and hypoxia (Shvetsova et al., 2017). And thus, both *HIF1AN and MPV17* have a negative effect on *HIF-1α.* It has been widely accepted that *HIF-1α* is a modulator in various organisms for sensing and responding to changes in the oxygen concentration, mediating the cellular adaptation to hypoxia (Menendez-Montes et al., 2016; Zhang et al., 2019). The expression of *HIF1* is essential to vasculogenesis and ventricular development at early gestational stage under hypoxic conditions. However, sustained *HIF1* activation at midgestation precludes regulation of genes essential for establishment of the cardiac conduction system, such as genes encoding gap-junction protein Connexin 40 essential for the rapid cardiac conducting capacity, and also impedes energy metabolism in mitochondrion, accompanied by increased oxidative stress, resulting in misregulation of genes involved in cardiac conduction system maturation (Menendez-Montes et al., 2016). *HIF-1α* protein expression is elevated in AF atrial tissues *in vivo* than those with SR (Ogi et al., 2010). *HIF-1α* can increase the expression of matrix metalloproteinases (MMPs) and transforming growth factor (TGF)-β in AF through promoting atrial fibrosis (Ogi et al., 2010), and inhibiting the expression of *HIF-1α* can decrease the levels of TGF-β and MMP-9 accompanied by the less myocardial fibrosis in rabbit models (Su et al., 2014). *HIF-1α* was found increased in LAA of patients with AF, suggesting that *HIF* is involved in the inflammatory and fibrotic change of epicardial adipose tissue (Abe et al., 2018). Furthermore, reduced fatty acid oxidation caused by upregulation of *HIF-1α* has been demonstrated in AF models (Bai et al., 2019). *HIF1AN* could be a potential strategy for limiting exacerbated inflammation responses (Palazon et al., 2014). Increased *HIF1AN* in patients with AF in our bioinformatics-based findings manifested the protective property of *HIF1AN* against atrial fibrosis and inflammation induced by *HIF-1α* in AF.

GSEA can be used to estimate changes in pathway activity in a sample population (Xie et al., 2022). Using GSEA, we found that high expression of *MPV17* was significantly enriched in GAP JUNCTION pathway. Gap junctions in myocardium are responsible for the intercellular conduction of action potentials; Spach et al. suggested that altered topology of gap junctions took important effects on AF and could be used as a therapeutic strategy for AF (Spach and Starmer, 1995). In recent years, the concept of AF treatment with a normalized gap junction distribution has been widely recognized. It is now believed that various mechanisms affecting gap junctions could lead to an increased susceptibility to AF. Gap junction remodeling during AF can be reversed, which can be accompanied by a decrease in atrial susceptibility to AF (Guo and Yang, 2022). GSEA also revealed that high *HIF1AN* expression was significantly enriched in the inositol phosphate metabolism pathway. This pathway, in which extracellular signaling molecules are activated by binding to cell surface G protein-coupled receptors to release phosphatidylinositol, possibly influences the regulation of cardiac function, especially during the post-ischemic reperfusion of the myocardium (Anderson et al., 1995). The cAMP produced in the activation of phosphatidylinositol metabolism, such as inositol-1,4,5-triphosphate receptors (InsP3Rs), modulates atrial muscle contraction and is thought to contribute to AF. The expression levels of InsP3Rs are upregulated in patients with AF—a process that may be associated with Ca^2+^ release and altered homeostasis (Varma et al., 2022). These findings suggest that these signaling pathways are involved in the onset and development of AF and that *MPV17* and *HIF1AN* may be involved in the pathogenesis of AF through these signaling pathways.

During AF, the immune system is greatly altered and plays an important role in the pathophysiology of disease development (Yao et al., 2022). We found neutrophils, mast cells, NK cells, and IFN-γ to be significantly elevated in AF and Th2 cells to be significantly decreased among 22 types of immune cell. The number of neutrophilic granulocytes was higher in the atrial fat tissue of AF patients relative to SR individuals (Begieneman et al., 2015). Neutrophils play an important role in AF development by releasing cytokines such as interleukin-6 (IL-6), tumor necrosis factor-α(TNF-α), MMP-2 and ROS that promote atrial remodeling (Liu et al., 2018). Mast cells get involved in cardiac fibrosis by releasing fibrogenic mediators in animal models of AF (Kong et al., 2014; Uemura et al., 2016). However, the number of mast cells in AF patients was similar to those with SR (Smorodinova et al., 2017). The controversial effect of mast cells in AF is due to some anti-fibrotic mediators in mast cells (Yao et al., 2022). The influence of Th2 cells on AF is uncertain. Anti-inflammatory cytokines (IL-4 and IL-10) secreted by Th2 cells downregulate cell-mediated immune responses and cytotoxic inflammatory responses. However, Th2 cells have also been implicated in the pathogenesis of fibrotic conditions (Liu et al., 2018). IFN-γsecreted by T cells and NK cells promotes atrial remodeling through macrophage (Liu et al., 2015; Liu et al., 2018).

Hypoxia delays neutrophil apoptosis through HIF-1α-dependent nuclear factor-κB activity that results in sustained inflammation (Walmsley et al., 2005). The functions of the HIF on macrophage polarization depend on the pathophysiological context. *HIF-1α* can also drive immunosuppressive functions by macrophages lacking *HIF-2α* fail to mount an inflammatory response (Palazon et al., 2014). Hypoxia induces the production of proinflammatory cytokines (TNF-α, IL-1β) by dendritic cells (DCs) (Mancino et al., 2008). Due to the inhibitary effects of *HIF1AN* and *MPV17* on *HIF-1α* (Mahon et al., 2001; Shvetsova et al., 2017), the hub genes attenuate the influence of immune response induced by *HIF-1α*. In our bioinformatics-based findings, *HIF1AN* had a positive relationship with NK cells and macrophages, and a negative relationship with DCs in AF; *MPV17* had a positive association with B cells in AF.

Although the hub genes screened based on WGCNA and the machine learning methods showed accurate diagnostic power and were validated using external datasets, there were still some limitations should be announced. First, this study was based on bioinformatics analyses to obtain hub genes, it is necessary to be cautious to conclude gene expression elucidating the molecular mechanisms underlying AF without verification *in vivo* and *in vitro* experiments, and thus, further investigation is warranted to address *in vitro* and *in vivo*. Second, external clinical features of these data were not used in our study. Furthermore, immune cell infiltration analysis was based on limited genetic data, and the specific regulatory mechanisms need further *in vivo* and *in vitro* experimental validation.

## 5 Conclusion

In summary, we used WGCNA combined with machine learning methods to screen and validate the hub genes and identified the correlations between two hub genes and the immune microenvironment and immune regulation. Our results may provide more accurate and effective diagnosis biomarkers and therapeutic targets for AF.

## Data Availability

The original contributions presented in the study are included in the article/Supplementary Material, further inquiries can be directed to the corresponding author.

## Abbreviations

AF, Atrial fibrillation; AUC, Area under the receiver operating characteristic curve; DC, Dendritic cell; DEG, Differentially expressed gene; GEO, Gene expression omnibus; GO, Gene ontology; GSEA, Gene set enrichment analysis; GSVA, Gene set variation analysis; GWAS, Genome-wide association study; HIF-1α, Hypoxia inducible factor 1-alpha; IFN, Interferon; IL, interleukin; LASSO, Least absolute shrinkage and selection operator; MMP, Matrix metalloproteinase; NES, Normalized enrichment score; NK, Natural killer; ROC, Receiver operating characteristic; ROS, Reactive oxygen species; SVM-RFE, Support vector machine based recursive feature elimination; SR, Sinus rhythm; TGF, Transforming growth factor; Th T, helper; TNF, Tumor necrosis factor; TOM, Topological overlap matrix; WGCNA, Weighted gene co-expression network analysis.
