# Supplementary figures and images for: Identification and validation of potential biomarkers for atrial fibrillation based on integrated bioinformatics analysis
Source: Front Cell Dev Biol. 2024 Jan 11;11:1190273. doi: 10.3389/fcell.2023.1190273 (PMC10808641; doi:10.3389/fcell.2023.1190273)

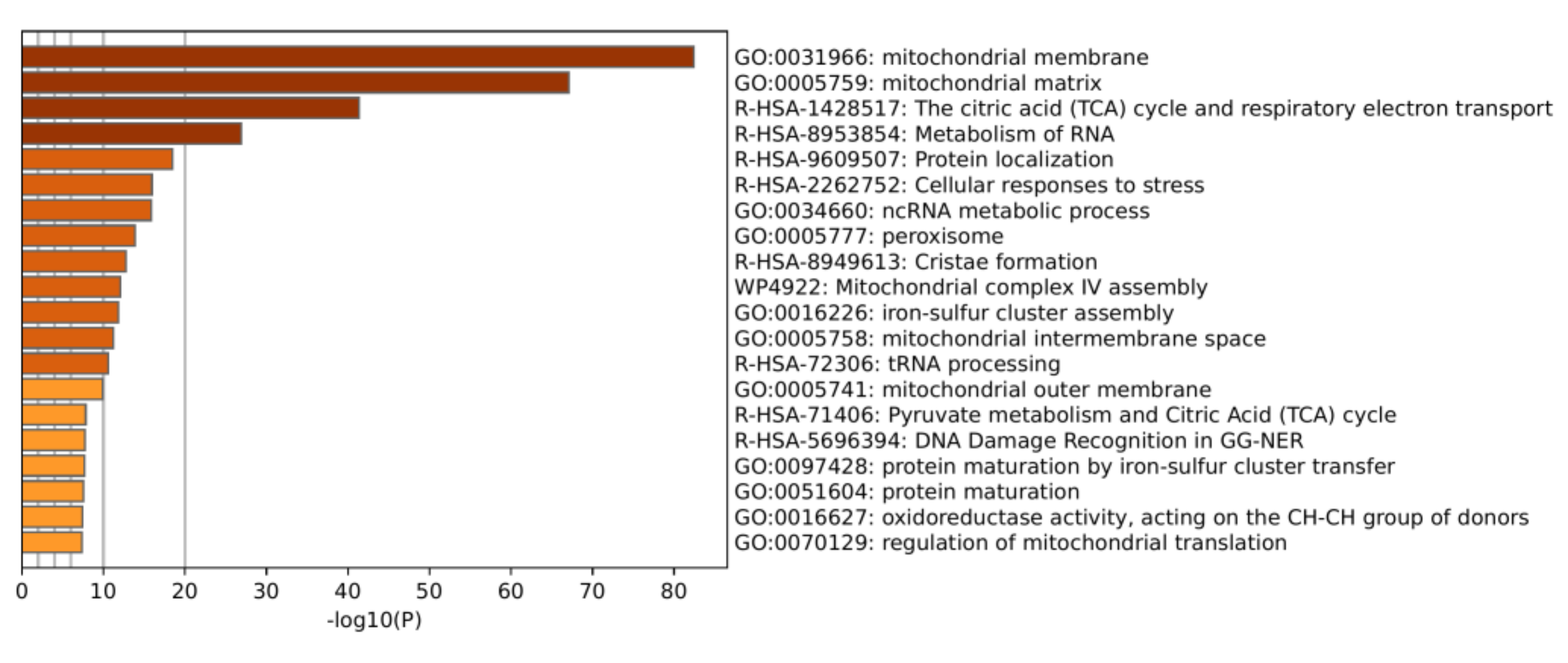

Supplement: Supplementary file 1 [file Image1.TIFF]

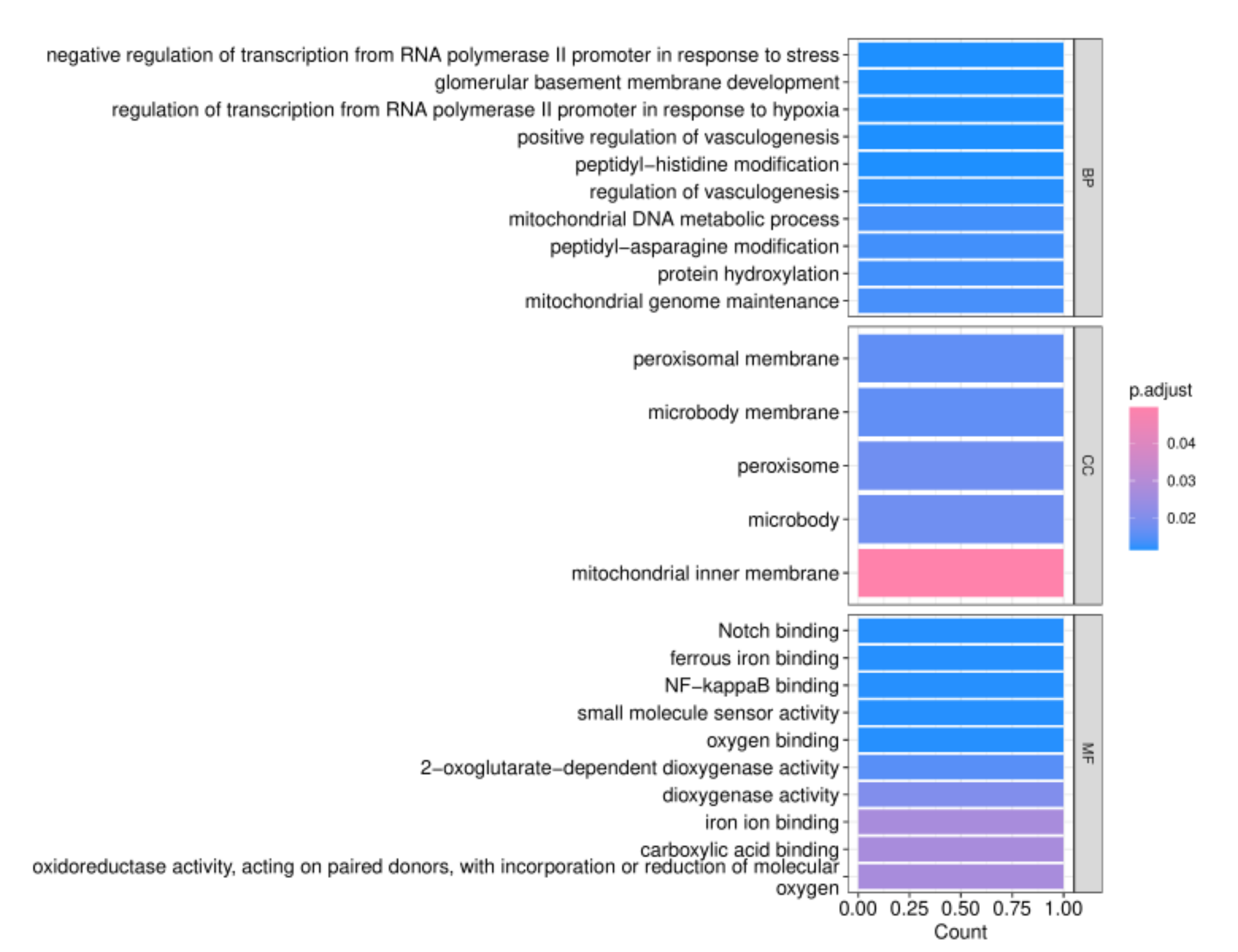

Supplement: Supplementary file 2 [file Image2.TIFF]
